# Abstract Knowledge in the Broken-String Problem: Evidence from Nonhuman Primates and Pre-Schoolers

**DOI:** 10.1371/journal.pone.0108597

**Published:** 2014-10-01

**Authors:** Carolina Mayer, Josep Call, Anna Albiach-Serrano, Elisabetta Visalberghi, Gloria Sabbatini, Amanda Seed

**Affiliations:** 1 University of St Andrews, School of Psychology and Neuroscience, St Andrews, Fife, Scotland, United Kingdom; 2 Max Planck Institute for Evolutionary Anthropology, Department of Comparative and Developmental Psychology, Leipzig, Germany; 3 Ethology and Animal Welfare Section, Universidad CEU Cardenal Herrera, Valencia, Spain; 4 Unit of Cognitive Primatology and Primate Centre, Istituto di Scienze e Tecnologie della Cognizione, Consiglio Nazionale delle Ricerche, Roma, Italy; University of Padova, Italy

## Abstract

There is still large controversy about whether abstract knowledge of physical problems is uniquely human. We presented 9 capuchin monkeys, 6 bonobos, 6 chimpanzees and 48 children with two versions of a broken-string problem. In the standard condition, participants had to choose between an intact and a broken string as means to a reward. In the critical condition, the functional parts of the strings were covered up and replaced by perceptually similar, but non-functional cues. Apes, monkeys and young children performed significantly better in the standard condition in which the cues played a functional role, indicating knowledge of the functional properties involved. Moreover, a control experiment with chimpanzees and young children ruled out that this difference in performance could be accounted for by differences of perceptual feedback in the two conditions. We suggest that, similar to humans, nonhuman primates partly rely on abstract concepts in physical problem-solving.

## Introduction

Adult humans rely on the abstract representation of objects’ physical properties in their daily problem-solving. Although several other animal species can use tools to solve problems [1], [2], the nature of their object representations is a matter of intense debate. For example, having learned to pull an intact object rather than a broken one to bring food within reach, chimpanzees, capuchin monkeys and cotton-top tamarins transferred the solution across tasks that varied the tools’ shape and position [3]–[9]. It is possible that the primates have abstract knowledge of object properties such as connectivity or continuity [5], [6]. Alternatively, however, they might have generalised the solution using a perceptual metric common to all of the tasks (e.g. avoid a gap between two parts of a tool). It is difficult to tease apart these two explanations based only on successful transfers, since both strategies would enable subjects to solve all problems in which the same perceptual features are discriminatory [9]. To date, the notion that any non-human animals go beyond perceptual features of objects to represent their abstract physical properties remains contentious [10], though see [11], [12].

We aimed to overcome these limitations by comparing the performance of bonobos, chimpanzees and tufted capuchin monkeys on two versions of a broken-string problem. All three species are known to solve a variety of tasks that require them to discriminate between two or three tools [3], [13]–[17]. We also tested children between 2 ½ and 6 ½ years of age to gain insight into the development of using object properties and arbitrary cues to solve problems.

In the standard, ‘uncovered’ version of the task, a reward was tied to each of two strings, one complete and one broken in two parts with a gap between the two. In the ‘covered’ version, the central parts of the strings were obscured by a cover; although the rewards at the far end of the strings were visible. A broken and an unbroken string were stuck to the cover in the same positions as their real counterparts beneath.

**Figure 1:**
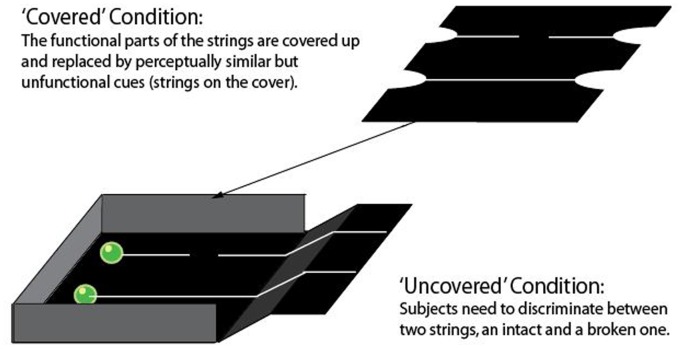
Apparatus for the ‘covered’ and the ‘uncovered’ condition. In the ‘covered’ condition, the functional parts of the strings were covered up with a cover. The perceptually identical, but non-functional strings on the cover represented their real counterparts underneath. The rewards could still be seen at the same distance as in the ‘uncovered’ condition. Only the object movements were obscured in the ‘covered’ condition.

The ‘covered’ and the ‘uncovered’ conditions were perceptually very similar. A subject could use the appearance of the strings (i.e., with and without a gap) to choose (pull or touch) the correct alternative (the string that could bring the food into reach) (see figure 1). Moreover, in both conditions, the movement of the reward at the end of the table could immediately be perceived by subjects choosing the unbroken string, so there was similar visual reinforcement. Therefore, relating perceptual features to the outcome would lead subjects to solve both conditions equally well. However, the physical connection between the unbroken string and the reward could only be seen in the ‘uncovered’ condition; as the strings moved after they had been chosen. In contrast, the cues on the cover in the ‘covered’ condition did not move.

This approach allowed us to test the hypothesis that nonhuman primates rely on perceptual cues alone when discriminating between two tools in a means-end task: if subjects only respond to the perceptual pattern of the problem, they should perform equally well in both the ‘covered’ and ‘uncovered’ condition. In contrast, if subjects have knowledge of the functional properties of the problem (i.e., the gap in the string prevents you from pulling in the reward), one would predict better performance in the ‘uncovered’ condition in which the strings play a functional role.

## Experiment 1: Nonhuman Primates

### Participants

We tested 6 chimpanzees (4 females) and 6 bonobos (3 females) at the Wolfgang Köhler Primate Research Centre (WKPRC) at the Leipzig Zoo, Germany, between March and August 2008 (age range = 3–31 years). Chimpanzees at the WKPRC live in two cohesive groups ranging from 6 to 17 individuals. The groups are housed in separate ∼4000 m^2^ outdoor areas, and ∼400 m^2^ indoor areas, which have natural vegetation, climbing structures, trees, streams and other natural features, as well as enrichment facilities such as spinning treat logs and artificial termite mounds. At night they stay in a series of sleeping rooms (about 47 m^2^). The chimpanzees are fed a variety of fruits, vegetables and cereals several times per day. The subjects are never food deprived and water is available ad libitum. Individual ages can be found in S1. All of the apes had previous experience with a number of problems involving tools (including strings); both in their enriched captive environment, as well as in previous experiments. Chimpanzees were tested individually in an on-show observation room, and bonobos in their sleeping rooms. Subjects were tested on consecutive days as far as possible, with no more than 7 days between testing days. Research at the WKPRC was performed in accordance with the recommendations of the Weatherall report “The use of non-human primates in research”. Research was non-invasive and strictly adhered to legal requirements in Germany. The study was ethically approved by an internal committee at the Max Planck Institute for Evolutionary Anthropology. Animal husbandry and research comply with the “EAZA Minimum Standards for the Accommodation and Care of Animals in Zoos and Aquaria”, the “WAZA Ethical Guidelines for the Conduct of Research on Animals by Zoos and Aquariums” and the “Guidelines for the Treatment of Animals in Behavioral Research and Teaching” of the Association for the Study of Animal Behavior (ASAB).

We also tested 9 capuchin monkeys (4 females) at the Primate Center of the Institute of Cognitive Sciences and Technologies, CNR in Rome (Italy) between February and June 2013 (age range = 3–29 years). Capuchin monkeys at the CNR are held in four separated groups ranging from 5 to 11 individuals. The groups are housed in enclosures consisting of an outdoor area ranging from ∼19.4 m2 to ∼126.8 m2 with natural vegetation and climbing structures and two ∼8.7 m2 indoor cages. Similar to the apes, none of the monkeys are ever food deprived, but fed monkey chow (Altromin-A pellets, Rieper standard diet for primates), fruits and vegetables each day according to their diet. Water is available ad libitum. Individual ages can be found in S1. All capuchin monkeys had previous experience with a number of problems involving tools (but not with string tasks). Capuchins were tested individually in the indoor area. Testing took place a maximum of 5 times a week, with no more than 7 days between testing days. All procedures at the CNR complied with the protocols approved by the Italian Health Ministry (Licence no. 12/2011-C) and were performed in full accordance with the European law on humane care and use of Laboratory animals. The experiments performed in our study adhere to the ASAB/ABS Guidelines for the use of Animals in Research.

### Material

The apparatus consisted of a table with two strings on top of it and was situated outside of the testing enclosure in front of a Perspex panel that was attached to the mesh. The apes’ table was made of 1 cm thick, black painted Perspex, (72 cm × 78 cm) and the table for the capuchin monkeys was made of 0.5 cm black painted wood (32.5 cm × 31.5 cm). The different proportions of the tables (and other materials involved) were adjusted to the differences in body size of apes and capuchins. We used 0.5 cm diameter white, plastic-coated nylon strings that were 92 cm- long for the apes, and 35 cm- long for the monkeys. The strings were placed in front of two holes in the Perspex panel separated 36 cm for the apes and 20 cm for the monkeys; the holes were 7 cm diameter for the apes and 3.5 cm diameter for the monkeys. For the apes, a device was attached in front of the holes that constrained subjects to one choice at a time – if subjects moved the device to open one hole, the other one was closed. Then the experimenter removed the non-chosen string. Monkeys were restricted to one choice at a time by moving the apparatus back immediately after they started pulling one of the strings. Both apes and monkeys had to pull the string towards themselves in order to obtain a reward (if their choice was correct). The rewards were tied to the further ends of the strings; these were pieces of banana, monkey chow, or whole grapes for the apes, depending on the individual preferences, and small peanut pieces for the monkeys. The strings were placed on top of double-sided tape so that they lay straight on the table. When pulled, the string would come away from the tape easily. The gap in the non-functional option was 5 cm long for all subjects. This was done to ensure that the salience of the gap was held constant for all nonhuman primates. For the apes it was either close to the subject (32 cm), far from the subject (58 cm) or in the middle of the string (45 cm). This was to investigate the role of split-attention between the reward and the gap. The variation in gap position had no effect on performance (S2); therefore, it was not used for the capuchin monkeys and it is not discussed further.

In the ‘covered’ condition, the functional parts of the strings were obscured with a cover of the same material as the apparatus. This cover was placed over the functional strings at a height that just allowed the reward to pass freely beneath it and two half-circles were cut into the cover (see fig. 1) to allow the subject to see the rewards at the far-end of the table. Non-functional strings of the same material and size as in the ‘uncovered’ condition were stuck on the cover coinciding exactly with the functional strings below.

### Procedure

Half of the subjects started on the ‘covered’ and half started on the ‘uncovered’ version of the problem. The experimenter positioned the baited strings behind an occluder before raising it and allowing the subject to make a choice. As soon as the subject pulled or touched one string, the experimenter removed the other string. Testing was interrupted if subjects refused to participate and was continued on the next testing day. If they scored 11 or 12 correct on the first day of testing, they moved to the next condition. If they did not, they continued to another 12 trials until they reached the criterion of 19 out of 24 or more, or until 120 trials had been completed. In both cases, participants then moved to the next condition. This success criterion ensured that subjects had chosen the correct string significantly above chance (α-level = 0.01; we ran binomial tests to calculate the p-value for 11 correct choices out of 12, *p* = 0.006 and 19 correct choices out of 24, *p* = 0.007) and was used for both conditions of all experiments. For all subjects the correct choice was presented equally often on the left and on the right side of the apparatus within a session. This was done in a pseudorandom, pre-prescribed order, with the restriction that no more than 2 trials on one side were given consecutively, to discourage side biases. The percentage of correct trials for each condition was analysed. Trials were scored live on a coding sheet and recorded on mini DV tape.

#### Transfer

To exclude the possibility that apes and monkeys preferred the longer string, we conducted an ‘uncovered’ transfer task for successful subjects in which the correct choice was always shorter than the first part of the broken string. A maximum of 36 trials was conducted and criterion was identical to the test-phase (i.e. 11 or more correct choices out of 12 on the first day of testing, and 19– or more- out of 24 thereafter).

### Results

**Figure 2:**
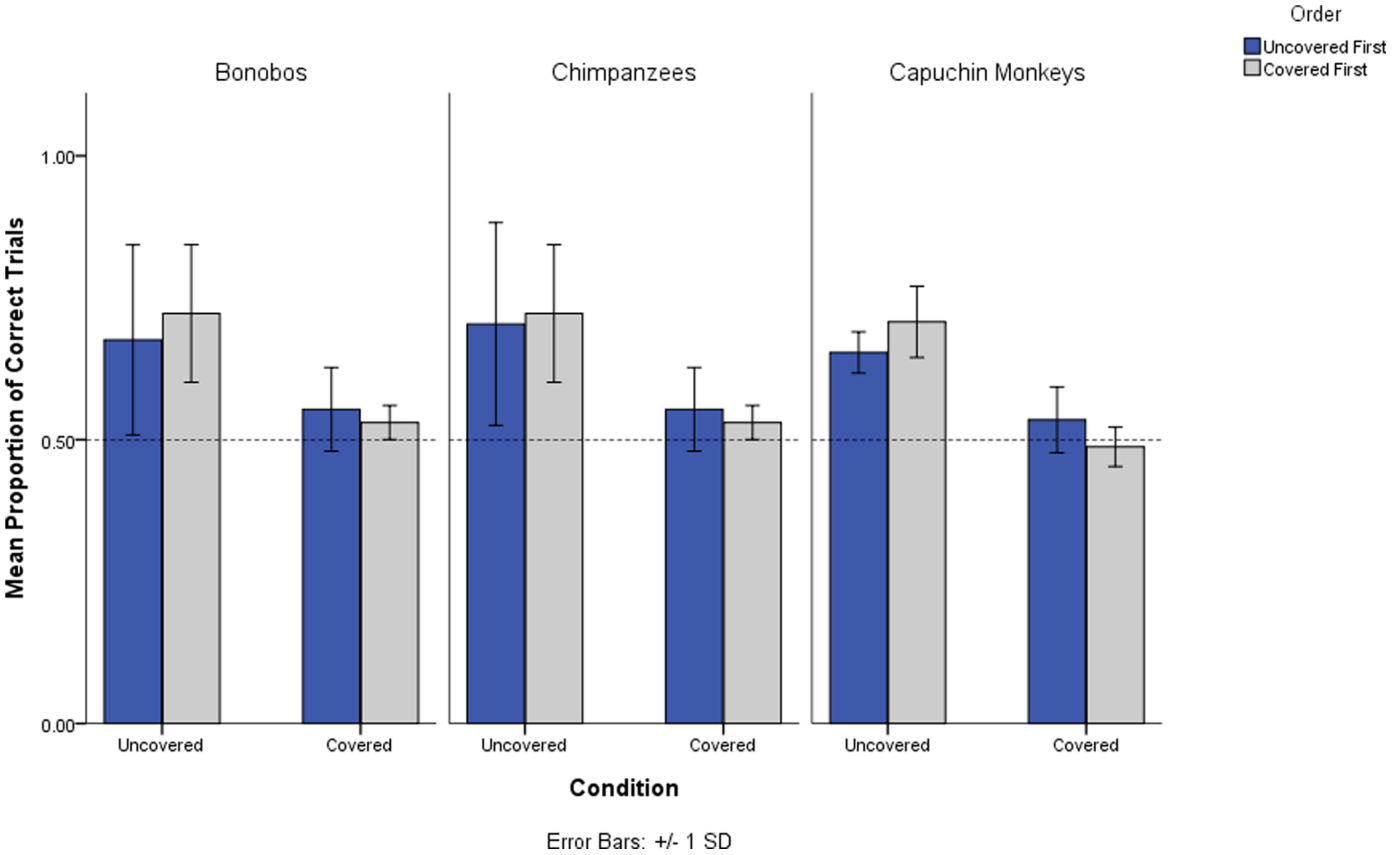
Proportion of correct trials for chimpanzees, bonobos and capuchin monkeys in the ‘covered’ and ‘uncovered’ condition. Dark bars represent the ‘uncovered-first’ group and light bars the ‘covered-first’ group.

The performance of great apes and capuchin monkeys did not differ across the two conditions in experiment 1; however, both species performed significantly poorer in the ‘covered’ condition compared to the ‘uncovered’ condition. In line with previous studies [3], [12]–[17], eight out of 12 great apes and six out of eight capuchin monkeys quickly solved the ‘uncovered’ condition. All three species required a similar amount of trials to reach criterion in the ‘uncovered’ condition (chimpanzees, *M _Trials to criterion_* = 64; bonobos, *M _Trials to criterion_* = 75; capuchin monkeys, *M _Trials to criterion_* = 80), with no significant species differences, (*F*_(2,18)_ = .36, *p*>.05). The proportion of correct trials for the ‘uncovered’ and ‘covered’ was normally distributed, Kolmogorov-Smirnov-test, ‘uncovered’, *Z* = .69, *p*>.05; ‘covered’, *Z* = .74, *p*>.05. Thus, the proportion of correct trials for each individual was analysed in a mixed-model ANOVA with condition as a within-subjects factor, and species and order of task presentation as between-subjects factors. There was no significant effect of species (*F*_(2,15)_ = .44, *p*>.05) nor order of task presentation on performance (*F*_(1,15)_ = .00, p>.05), and no significant interactions. However, the ANOVA revealed a significant effect of condition on performance, (*F*_(1,15)_ = 30.13, *p*<.01), with no significant interactions (see figure 2 and S1). All ape and monkey subjects successfully solved the string-length transfer within 36 trials or less (i.e. all primates reached criterion within 36 trials).

### Discussion

The difference in performance between the ‘covered’ and the ‘uncovered’ conditions suggests that nonhuman primates’ ability to choose the unbroken string is not based on simply associating the outcome with a response to a perceptual cue. In fact, none of the subjects solved the ‘covered’ condition, regardless of whether they received this task first or second. Strikingly, even successful subjects of the ‘uncovered’ first-group did not use the perceptual information given by the cues on the cover to discriminate between the options in the ‘covered’ condition, despite the close perceptual similarity to the cues given in the ‘uncovered’ condition. We suggest that subjects benefitted from the combination of perceptual and functionally-relevant information when they learned to solve the ‘uncovered’ condition. However, removing visual access to the functional gap also restricts visual access to the movement of the strings, and this difference in perceptual feedback could be an alternative reason for the difference in performance. We address this alternative in Experiment 3.

## Experiment 2: Children

### Participants

Children from 10 kindergartens across Leipzig were tested between November 2008 and April 2009∶12 in each of 4 age-groups: 2 ½, 3 ½, 5 ½ and 6 ½ year-olds. We did not test 4 ½ year-olds due to time constraints. In each age group we tested equal numbers of boys and girls. Children were tested individually in a small room near their classroom. Subjects were tested on consecutive days as far as possible, with no more than 3 days between testing days. We used a window of 2 months above and below the target age. A further 9 children were dropped from the study due to experimenter's error (6) or because they did not complete all phases of testing, either because they went on holiday (2) or became ill (1). The study was ethically approved by an internal committee at the Max Planck Institute for Evolutionary Anthropology. There is documented informed consent from parents/guardians.

### Material

The apparatus for the children was made of cardboard, ‘covered’ in blue sticky, backed plastic (101 cm × 61 cm) and placed on the floor, with the child sitting behind a 60 cm × 60 cm thick Perspex window fixed to the front of it. The strings were 121 cm long white wool and lay 32 cm apart, in front of two 7 cm diameter holes in the Perspex panel. As for the apes, a device was attached to the front of the panel that constrained subjects to one choice at a time. The 5 cm gap in the broken string was either close to the subject (45 cm), far from the subject (71 cm) or in the middle of the string (58 cm). We found a significant effect of gap position on performance, but as our main results remained unaffected, the latter finding is not discussed further (see S2). The rewards were clear plastic balls containing stickers. Similar to the apes and monkeys, in the ‘covered’ condition the functional parts of the strings were covered with a cover of the same material as the apparatus. Again, this cover was placed over the real strings at a height that just allowed the reward to pass freely beneath it. Non-functional strings were stuck on the cover with the same materials and measures as in the ‘uncovered’ condition.

### Procedure

The experimental procedure was identical to the apes and monkeys and contained only minimal verbal instructions (i.e. “Try to get a sticker!”) with the only difference that children received 24 trials per day and a maximum of 48 trials per condition. The success criterion for both conditions (‘covered’ and ‘uncovered’) was identical to that used in experiment 1.

### Results

**Figure 3:**
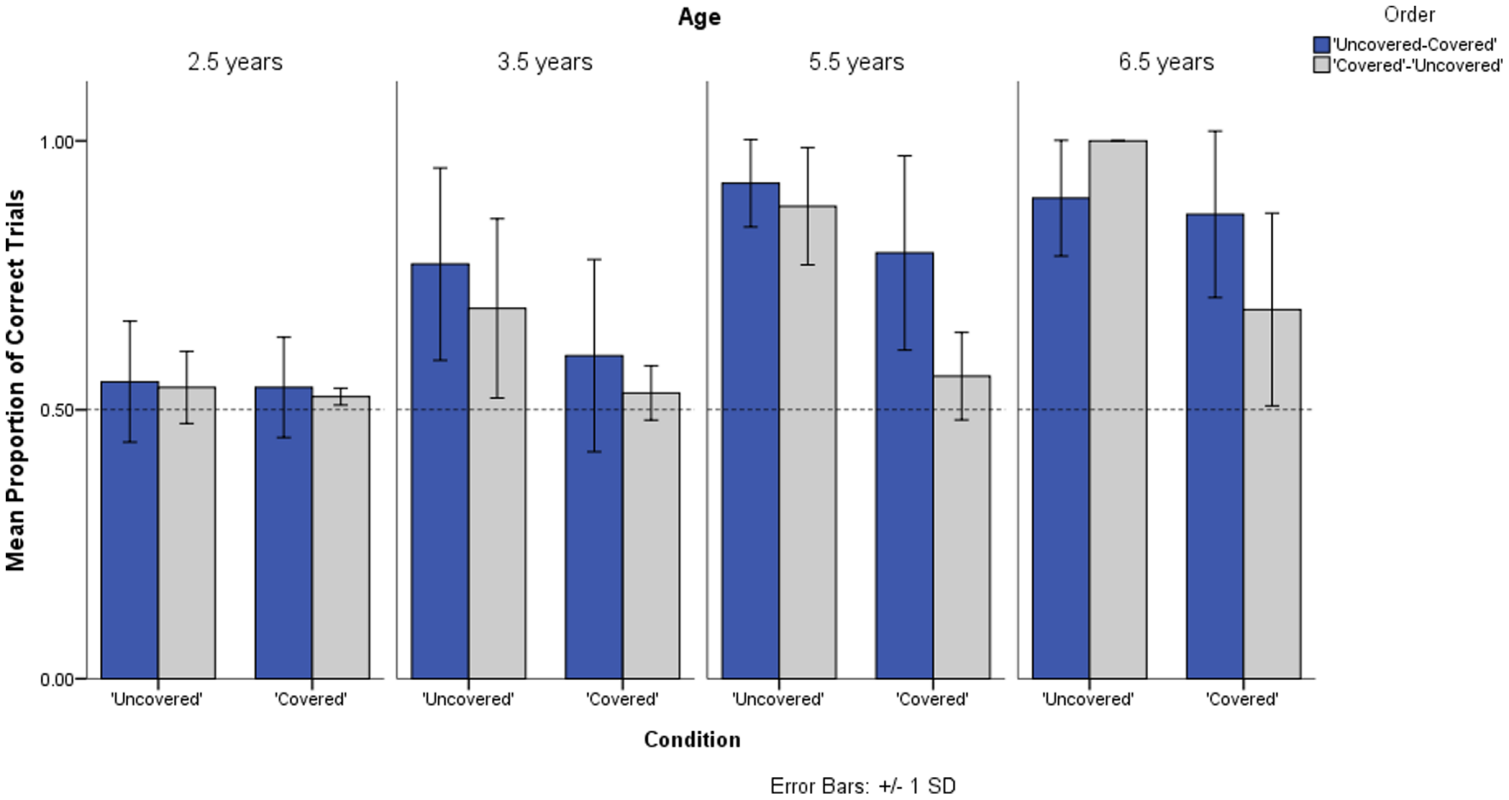
Proportion of correct trials for children of different age groups in the ‘covered’ and ‘uncovered’ condition. Dark bars represent the ‘uncovered-first’ group and light bars the ‘covered-first’ group.

Overall children of all age groups performed better in the ‘uncovered’ than in the ‘covered’ condition, but performance in the ‘covered’ condition improved with age (see figure 3). One of twelve 2 ½ year-olds, 8 of twelve 3 ½ year-olds (*M _Trials to criterion_* = 33), and all 5 ½ (*M _Trials to criterion_* = 22 trials) and 6 ½ year-olds (*M _Trials to criterion_* = 18 trials) reached criterion in the ‘uncovered’ version of the task. In contrast, only one of twelve 2 ½ year-olds; 2 of twelve 3 ½ year-olds (*M _Trials to criterion_* = 30 trials); 6 of twelve 5 ½ year-olds (*M _Trials to criterion_* = 26 trials) and 7 of twelve 6 ½ year-olds (*M _Trials to criterion_* = 26.7) reached criterion in the ‘covered’ task. The proportion of correct trials in the ‘uncovered’ and ‘covered’ were normally distributed, Kolmogorov-Smirnov-test, ‘uncovered’, *Z* = 1.29, *p*>.05; ‘covered’, *Z* = 1.32, *p*>.05. A mixed-model ANOVA (with condition as a within-subjects factor, and age and order of task presentation as between-subjects factors) on the proportion of correct trials in children revealed a significant three-way interaction between condition×age×order (*F*_(3,40)_ = 4.83, *p*<0.01) (see figure 3). A Scheffe posthoc test indicated that 5 ½ year-olds performed significantly better than 2 ½ year-olds and 3 ½ year-olds, all *p*’s <.01; as did 6 ½ year-olds, all *p*’s <.01. There were no significant differences between 5 ½ year-olds and 6 ½ year-olds, *p>*.05, or between 2 ½ year-olds and 3 ½ year-olds, *p>*.05. Thus, we collapsed the data of older children (5 ½ year-olds and 6 ½ year-olds) and the data of younger children (2 ½ year-olds and 3 ½ year-olds). Looking at these two groups separately, a mixed-model ANOVA in young children revealed a significant effect of condition (*F*_(1,22)_ = 5.1,8, *p*<.05) with no effect of order and no significant interactions. In fact, similar to apes and capuchins, most young children failed the ‘covered’ condition regardless of whether they received this condition first or second, but they performed well in the ‘uncovered’ condition. It has to be noted, however, that 2 ½ year-olds largely failed to reach criterion in both the ‘covered’ and ‘uncovered’ condition (Figure 3 and S3). In contrast, older children showed a significant interaction between condition and order (*F*_(1,22)_ = 15.18, *p*<.01), and a significant effect of condition *(F*_(1,22)_ = 42.83, *p*<.001). Performance in the ‘covered’ condition was better in the group that received the ‘uncovered’ condition first (*M _Proportion of correct trials_* = .83, *SD* = .16) compared to the group that received the ‘covered’ condition first (*M _Proportion of correct trials_* = .62, *SD* = .15).

### Discussion

The different performance in the ‘covered’ and ‘uncovered’ conditions indicates that, similar to apes and capuchins, 3 ½ year-old children did not learn to avoid the broken string by using only its appearance as an arbitrary cue. Instead, learning required both visual access and feedback related to the functional properties of the strings. This is consistent with previous work on the developmental trajectory of the use of object knowledge in children. ‘Core knowledge’ accounts of infant cognitive development posit that basic object concepts such as continuity and solidity emerge in the first months of life [18], but immature executive resources may prevent children from acting on their knowledge until around 3 years of age [19]. The finding that 2 ½ year-old children largely failed to solve the ‘uncovered’ condition, while most 3 ½ year-old children solved this condition might be similarly accounted for.

The poor performance of the two younger age groups in the ‘covered’ condition is surprising in light of other work. Gopnik et al [20], [21] found that 2–4 year-old children were able to learn a relationship between an arbitrary cue (blue, not red block) and an outcome (a sound activated by the experimenter surreptitiously) in very few trials. In our study, 3 ½ year-olds starting with the ‘covered’ condition did not learn to avoid the side with the gap on the cover (arbitrary cue). However, there is a clear difference between the tasks concerning the transparency of the mechanics involved. While the mechanics were deliberately opaque in Gopnik et al [20], [21], they were obvious in our study (pull a string to get a reward). It might be harder to relate an arbitrary cue with an outcome if the mechanics of a problem are known to the subject than if they are not. If capuchins, apes and children have knowledge about the relevant properties involved in pulling a string, then they might be less likely to view the perceptual cues present in the ‘covered’ condition as relevant to the problem’s solution.

Successful performance in the ‘covered’ version increased with age in the children. Unlike the apes and younger children, some 5 ½ and 6 ½ year-olds were able to solve the ‘covered’ condition even when they received it first, though they performed better if they received it second. One possibility is that learning from perceptual surface features emerges late in human development. However, as discussed above, in other contexts 3 to 5 year-old children can form new associations and even causal representations from perceptual cues. In addition, the fact that older age groups performed better in the ‘covered’ condition if they received the ‘uncovered’ condition first does not fit well with an explanation based on improvements in associative learning of arbitrary patterns, which would not benefit from previous experience with the functional properties of a given task. An alternative explanation for the developmental pattern in human children is that with age, children are increasingly able to use the stimuli in the covered condition as iconic symbols. Indeed, DeLoache and colleagues found that children begin to use symbolic representations in the absence of explicit verbal instruction at a similar age (5–7 years), albeit from a different paradigm [22].

In sum, both children of all age groups and non-human primates performed better if the perceptual features and their functional context where present (‘uncovered’ condition) than if the perceptual features were arbitrarily related to the outcome (‘covered’ condition). However, the two conditions also differed in the amount of visual feedback provided to the subject. In the ‘covered’ condition, once a string had been pulled the string and the reward disappeared underneath the cover and its movement was obscured; this reduced amount of visual feedback might have led to poorer performance. To rule out this possibility, we conducted a further experiment with chimpanzees and 3 ½ -year-olds. We could not test bonobos and capuchins because of a lack of subjects that had not already participated in Experiment 1.

## Experiment 3: Chimpanzees and Children

### Subjects

We tested 6 chimpanzees (5 females) at the WKPRC (age range = 7–15 years) and 12 3 ½ year-old children (6 girls) from 3 kindergartens in Leipzig. Both chimpanzees and children were tested in September 2011. None of them had taken part in the previous experiments described above. The chimpanzees had previously participated in experiments involving choosing between two tools, including those made from string. See the supplemental materials for more information on the chimpanzees’ ages, rearing histories and housing conditions.

### Materials and procedure

The same apparatus of the original experiment was used for the experiment for both apes and children with the only difference that there were no cues present on the cover.

Testing was conducted according to the same experimental procedure described in experiment 1. The crucial difference lay in the replacement of the ‘covered’ condition with a ‘memory’ version of the broken-string problem, while the ‘uncovered’ condition remained identical. In the ‘memory’ condition participants were shown the functional strings for 2–3 seconds, before these were covered up by a plain cover. Thus, they had knowledge of the strings before making a choice; however, there were no visual cues present at the time of the choice and, similar to the ‘covered condition’ the string movement was partially obscured. Half of the subjects started on the ‘uncovered’ condition while half of the subjects started on the ‘memory’ condition. Scoring and maximum amount of trials and success criterion were identical to experiment 1 for chimpanzees and experiment 2 for children.

### Results

Both children and chimpanzees solved the ‘memory’ condition (but most subjects failed to reach criterion when it was presented first). Four out of 6 chimpanzees reached criterion in the memory condition (*M _Trials to criterion_* = 45 trials), but only one chimpanzee solved this condition when it was presented first. In contrast, all chimpanzees solved the ‘uncovered’ condition (*M _Trials to criterion_* = 36 trials). Seven out of 12 children reached criterion in the ‘memory’ condition (*M _Trials to criterion_* = 27.4 trials), but only one child solved this condition when it was presented first. Six out of 12 children solved the ‘uncovered’ condition if it was presented first (*M _Trials to criterion_* = 20 trials), and one child solved it when it was presented second. The proportion of correct trials for the ‘uncovered’ and the ‘memory’ condition were normally distributed for both chimpanzees (Kolmogorov-Smirnov-test, ‘uncovered’, *Z* = .54, *p*>.05; ‘memory’, *Z* = .69, *p*>.05) and children (Kolmogorov-Smirnov-test, ‘uncovered’, *Z* = .63, *p*>.05; ‘memory’, *Z* = .49, *p*>.05). A mixed-model ANOVA on the proportion of correct trials (with condition as a within-subjects factor, and species and order of task presentation as between-subjects factors) revealed a significant interaction of condition and order of task presentation (*F*_(1,14)_ = 19.92, *p*<.01) and a significant effect of order (*F*_(1,4)_ = 8.06, *p*<.05), but no other main effects. A Bonferroni pairwise comparison showed that subjects who received the ‘uncovered’ condition before the ‘memory’ condition performed better in the ‘memory’ condition (*M _Proportion of correct trials_* = .88, *SE* = .04) than subjects who received the ‘memory’ condition first (*M _Proportion of correct trials_* = .53, *SE* = .04) (see S4 for individual performance).

### Discussion

Both chimpanzees and 3 ½ year-olds who received the ‘uncovered’ condition first performed better than subjects starting with the ‘memory’ condition. In fact, only one chimpanzee and one 3 ½ year-old solved the ‘memory’ condition when it was presented first. This order effect indicates that visual feedback (unrestricted in the ‘uncovered’ condition) played a role in the solution of the broken-string problem. This is not surprising in light of recent work showing the importance of visual feedback for the acquisition of a new solution in chimpanzees [23]. Nevertheless, poorer performance in the ‘covered’ condition of experiment 1 compared to the ‘uncovered’ condition cannot be solely ascribable to the fact that the movement of the real strings was obscured from view. Both apes and 3 ½ year-olds solved the ‘memory’ condition (particularly, if they received it second), which has similarly reduced visual feedback. One could argue that in the ‘uncovered’ condition subjects had learned to associate the string without a gap with the reward and they might have transferred this association in the ‘memory’ condition without understanding the functional properties involved. However, if this was the case why would subjects have been capable to transfer this association to the ‘memory’ condition in experiment 3, but not to the ‘covered’ condition in experiment 1? The only difference between the ‘covered’ and the ‘memory’ condition was the visual access to the functional parts of the strings before being covered up in the ‘memory’ condition. Thus, the functional information present in the ‘memory’ condition and in the ‘uncovered’ condition seems to have been crucial for success. Future studies should investigate the role of visual feedback on the performance of capuchins and bonobos in the two versions of the broken-string problem.

## General Discussion

Penn et al [24] have suggested that representing higher-order abstract concepts is a uniquely human ability (but see [25], [26]); however, the difference between human and nonhuman primates might not be that simple. The difference may lie in symbolic relationships in particular, rather than abstract concepts in general [11]. Our study suggests that perceptually-based heuristics are not a good candidate explanation for the ability of non-human primates to avoid a broken tool: they perform poorly when perceptual cues are stripped of their functional relevance, implying that they make use of the functionally-relevant information about object properties. Chimpanzees, bonobos and capuchin monkeys may be able to conceptualize object features that transcend perceptual commonalities if they are grounded in functional contexts.

Chimpanzees and 3 ½ year-olds could use the functional cues to solve the ‘memory’ task despite restricted visual feedback, but performance was better if they first had received the ‘uncovered’ condition in which both functional information and full perceptual access to the object movement was provided. This might reflect the fact that finding new solutions to physical problems involves not only background knowledge about object properties, but also an ability to use that information to predict how an object will behave when acted upon. The latter skill is important if feedback is restricted, as in our memory task, or if the problem is ‘ill-structured’ (i.e. rather than choosing between two pre-determined alternatives, a new action sequence needs to be produced, such as manufacturing a hooked tool). Finding an innovative solution to a new problem is something that chimpanzees and even older children find difficult: this need not imply that they lack the requisite object knowledge; but precisely what cognitive skills are involved is an unanswered question (see [27]; [28] for a review). Future research should explore how pre-existing representations of objects and their properties interact with online perceptual feedback from acting on those objects when tackling a new physical problem.

## Supporting Information

Materials S1 **Supplemental Information for Experiment 1.**
(DOCX)

Materials S2 **Analysis of Gap-position for Experiment 1 and 2.**
(DOCX)

Materials S3 **Supplemental Information for Experiment 2.**
(DOCX)

Materials S4 **Supplemental Information for Experiment 3.**
(DOCX)
