# Supplementary material for: Abstract Knowledge in the Broken-String Problem: Evidence from Nonhuman Primates and Pre-Schoolers
Source: PLoS One. 2014 Oct 1;9(10):e108597. doi: 10.1371/journal.pone.0108597 (PMC4182709; doi:10.1371/journal.pone.0108597)
Supplement: Materials S1 — Supplemental Information for Experiment 1. (DOCX) [file pone.0108597.s001.docx]

**S1:** Supplemental Information for Experiment 1

*Table 1. Subjects in the ‘Uncovered’ (U) and ‘Covered’(C) condition.*

| Group | Subject | Sex  (f, m) | Age  (years) | Rearing | Order | Number of Trials to criterion | |
| --- | --- | --- | --- | --- | --- | --- | --- |
|  |  |  |  |  |  | **U** | **C** |
| Chimpanzees | Patrick | m | 11 | Mother | U🡪C | 36 | - |
|  | Tai | f | 6 | Mother | U🡪C | 60 | - |
|  | Fraukje | f | 32 | Mother | U🡪C | 96 | - |
|  | Lome | m | 7 | Mother | C🡪U | 36 | - |
|  | Pia | f | 9 | Mother | C🡪U | 36 | - |
|  | Dorien | f | 28 | Mother | C🡪U | - | - |
| Bonobos | Joey | m | 25 | Hand | U🡪C | - | - |
|  | Kuno | m | 11 | Hand | U🡪C | 24 | - |
|  | Ulindi | f | 14 | Mother | U🡪C | 36 | - |
|  | Limbuko | m | 12 | Hand | C🡪U | 48 | - |
|  | Yasa | f | 11 | Mother | C🡪U | - | - |
|  | Louiza | f | 3 | Mother | C🡪U | - | - |
| Capuchins | Carlotta | f | 29 | Hand | U🡪C | - | - |
|  | Robot | m | 24 | Hand | U🡪C | 72 | - |
|  | Robin Hood | m | 12 | Mother | U🡪C | 72 | - |
|  | Rucola | f | 27 | Hand | U🡪C | 108 | - |
|  | Paprica | f | 18 | Mother | C🡪U | - | - |
|  | Pedro | m | 16 | Mother | C🡪U | 48 | - |
|  | Roberta | f | 13 | Mother | C🡪U | 84 | - |
|  | Sandokan | f | 13 | Hand | C🡪U | 36 | - |

*Note.* Missing data in the number of trials to criterion (-) resemble a failure of the individual to reach criterion within the maximum amount of trials (120).

All three species required a similar amount of trials to reach criterion in the ‘uncovered’ condition (chimpanzees, *M* = 64; bonobos, *M* = 75; capuchin monkeys, *M* = 82.5), with no significant species differences, (*F*_(2,17)_ = .43, *p* > .05).
