# Supplementary material for: Abstract Knowledge in the Broken-String Problem: Evidence from Nonhuman Primates and Pre-Schoolers
Source: PLoS One. 2014 Oct 1;9(10):e108597. doi: 10.1371/journal.pone.0108597 (PMC4182709; doi:10.1371/journal.pone.0108597)
Supplement: Materials S2 — Analysis of Gap-position for Experiment 1 and 2. (DOCX) [file pone.0108597.s002.docx]

**S2:** Analysis of Gap-position for Experiment 1 and 2

Chimpanzees in Experiment 1 were presented with a gap that was either close to the subject (32 cm), far from the subject (58 cm) or in the middle of the string (45 cm). We investigated the effect of variation of gap position on performance in a mixed-model ANOVA with gap-position and condition (‘uncovered’ – ‘covered’) as within-subjects factors and species and order of task presentation as between-subjects factors. There was no significant effect of gap-position (*F*_(2,7)_=1.58, *p* >.05) and no significant interactions.

Children in Experiment 2 were also confronted with varying gap-positions. The gap was either close to the subject (45 cm), far from the subject (71 cm) or in the middle of the string (58 cm). We investigated the effect of variation of gap position on performance in a mixed-model ANOVA with gap-position and condition (‘uncovered’ – ‘covered’) as within-subjects factors and age group and order of task presentation as between-subjects factors. We also found a significant effect of gap position (F_(2,39)_ = 3.66, *p* <.05) with no significant interactions. A posthoc pairwise comparison revealed that the only significant difference in performance was between the gap being close to the subject or in the middle of the string, *p* <.05. Children performed overall better when the gap was in the middle of the string (*M* = .71) than when the gap was close to them (*M* = .68). This might indicate that children’s attention was focused on the middle and back part of the strings rather than the closer ends. The role of attention in physical problem-solving could be potentially interesting for future research.
