# Supplementary material for: Abstract Knowledge in the Broken-String Problem: Evidence from Nonhuman Primates and Pre-Schoolers
Source: PLoS One. 2014 Oct 1;9(10):e108597. doi: 10.1371/journal.pone.0108597 (PMC4182709; doi:10.1371/journal.pone.0108597)
Supplement: Materials S3 — Supplemental Information for Experiment 2. (DOCX) [file pone.0108597.s003.docx]

**S3:** Supplemental Information for Experiment 2

*Table 1. Children in the ‘Uncovered’ (U) and ‘Covered’(C) Condition in Experiment 2.*

| Age-group | Sex  (f, m) | Order | Number of Trials to criterion | |
| --- | --- | --- | --- | --- |
|  |  |  | **U** | **C** |
| 2 ½ year-olds | f | U🡪C | - | - |
|  | f | U🡪C | - | - |
|  | f | U🡪C | - | - |
|  | m | U🡪C | 48 | - |
|  | m | U🡪C | - | 48 |
|  | m | U🡪C | - | - |
|  | f | C🡪U | - | - |
|  | f | C🡪U | - | - |
|  | f | C🡪U | - | - |
|  | m | C🡪U | - | - |
|  | m | C🡪U | - | - |
|  | m | C🡪U | - | - |
| 3 ½ year-olds | f | U🡪C | 24 | 12 |
|  | f | U🡪C | 24 | - |
|  | f | U🡪C | 12 | - |
|  | m | U🡪C | - | - |
|  | m | U🡪C | 24 | - |
|  | m | U🡪C | - | 48 |
|  | f | C🡪U | - | - |
|  | f | C🡪U | - | - |
|  | f | C🡪U | 36 | - |
|  | m | C🡪U | 24 | - |
|  | m | C🡪U | 36 | - |
|  | m | C🡪U | 24 | - |
| 5 ½ year-olds | f | U🡪C | 24 | 24 |
|  | f | U🡪C | 36 | - |
|  | f | U🡪C | 12 | 12 |
|  | m | U🡪C | 24 | 24 |
|  | m | U🡪C | 12 | 24 |
|  | m | U🡪C | 12 | 24 |
|  | f | C🡪U | 24 | - |
|  | f | C🡪U | 48 | - |
|  | f | C🡪U | 24 | - |
|  | m | C🡪U | 12 | - |
|  | m | C🡪U | 12 | 48 |
|  | m | C🡪U | 24 | - |
| 6 ½ year-olds | f | U🡪C | 24 | 12 |
|  | f | U🡪C | 12 | 12 |
|  | f | U🡪C | 12 | 48 |
|  | m | U🡪C | 12 | 12 |
|  | m | U🡪C | 48 | 12 |
|  | m | U🡪C | 36 | 36 |
|  | f | C🡪U | 12 | - |
|  | f | C🡪U | 12 | 48 |
|  | f | C🡪U | 12 | - |
|  | m | C🡪U | 12 | 36 |
|  | m | C🡪U | 12 | - |
|  | m | C🡪U | 12 | 24 |

*Note.* Missing data in the number of trials to criterion (-) resemble a failure of the individual to reach criterion within the maximum amount of trials (120).
