# Supplementary material for: Abstract Knowledge in the Broken-String Problem: Evidence from Nonhuman Primates and Pre-Schoolers
Source: PLoS One. 2014 Oct 1;9(10):e108597. doi: 10.1371/journal.pone.0108597 (PMC4182709; doi:10.1371/journal.pone.0108597)
Supplement: Materials S4 — Supplemental Information for Experiment 3. (DOCX) [file pone.0108597.s004.docx]

**S4:** Supplemental Information for Experiment 3

*Table 1. Chimpanzees and Children in the ‘Uncovered’ (U) and ‘Memory’ (M) condition.*

| Group | Subject | Sex  (f, m) | Age  (years) | Rearing | Order | Number of Trials to criterion | |
| --- | --- | --- | --- | --- | --- | --- | --- |
|  |  |  |  |  |  | **U** | **M** |
| Chimpanzees | Trudi | f | 15 | Mother | M🡪U | 60 | 24 |
|  | Annette | f | 9 | Mother | M🡪U | 24 | 24 |
|  | Fifi | f | 15 | Mother | M🡪U | 48 | 24 |
|  | Alexandra | f | 9 | Mother | U🡪M | 24 | - |
|  | Jahaga | f | 15 | Mother | U🡪M | 36 | 108 |
|  | Alex | m | 7 | Mother | U🡪M | 24 | - |
| 3 ½ year-olds |  | f |  |  | M🡪U | 24 | - |
|  |  | f |  |  | M🡪U | - | - |
|  |  | f |  |  | M🡪U | 12 | - |
|  |  | m |  |  | M🡪U | - | - |
|  |  | m |  |  | M🡪U | - | - |
|  |  | m |  |  | M🡪U | 12 | 36 |
|  |  | f |  |  | U🡪M | 24 | 36 |
|  |  | f |  |  | U🡪M | - | 12 |
|  |  | f |  |  | U🡪M | - | 48 |
|  |  | m |  |  | U🡪M | 24 | 12 |
|  |  | m |  |  | U🡪M | - | 24 |
|  |  | m |  |  | U🡪M | 24 | 24 |

*Note.* Missing data in the number of trials to criterion (-) resemble a failure of the individual to reach criterion within the maximum amount of trials (120).
